# Experimental and Numerical Analysis of Multi-Hole Orifice Flow Meter: Investigation of the Relationship between Pressure Drop and Mass Flow Rate

**DOI:** 10.3390/s20247281

**Published:** 2020-12-18

**Authors:** Adam Tomaszewski, Tomasz Przybylinski, Marcin Lackowski

**Affiliations:** Institute of Fluid Flow Machinery, Polish Academy of Sciences, Fiszera 14 st., 80-231 Gdańsk, Poland; tprzybylinski@imp.gda.pl (T.P.); mala@imp.gda.pl (M.L.)

**Keywords:** multi-hole orifice, flow measurement, numerical simulation, pressure drop

## Abstract

The paper presents the results of the experimental and numerical analysis of a six-hole orifice flow meter. The experiments were performed on humid air in a 100 mm diameter duct. The aim of this research was to investigate the mass flow and pressure drop dependency in an orifice of a predetermined shape and to compare the results obtained with computational formulas recommended in the ISO 5167-2 standard for a single-hole orifice flow meter. The experiments and calculations were performed on several multi-hole orifice geometries with different contraction coefficient in a wide range of Reynolds numbers. The pressure was probed immediately upstream and downstream of the orifice. The flow coefficient determined for the six-hole orifice flow meter investigated was compared with the flow coefficient of conventional single-hole orifice with the same contraction coefficient. The results from computational formulas for single-hole orifice from ISO 5167 are also included in the paper. During some experiments, an obstacle has been introduced in the duct at variable distance upstream from the orifice. The effect of the thus generated velocity field disturbance on the measured pressure drop was then investigated. Numerical simulation of the flow with the presence of the obstacle was also performed and compared with experimental data.

## 1. Introduction

Accurate fluid mass flow measurement is required in many industrial applications. Common flow measuring devices such as the orifice meter, venturi meter and nozzle meter use pressure drop for determining the mass flow rate. One of the most popular types of flow meter is the single-hole orifice (SHO) meter. In ISO 5167-2 standard there is presented computational procedure [1,2] for mass flow determination for this kind of orifice. Such devices are characterized by simplicity of the design, reliability (no moving parts) and low manufacture cost. They have been standardized and approved for measurements relating to financial clearings. A conventional orifice has a single circular opening made in the center of a disc, which is usually mounted inside a pipeline. Recently, consideration has been given to application of multi-hole orifices (MHOs) for measuring purposes [3,4,5,6,7]. The metering principle is the same as for the standard orifice. However, compared to the standard orifice, the flow field parameters in the vicinity of the multi-hole orifice are more uniformly distributed across the entire pipe cross-section. All holes in the multi-hole orifice usually have the same hydraulic diameter and the minimum flow area depends on the β ratio [8]. The main advantage of a multi-hole orifice is better resistance to flow disturbance [5,9], which should result in a shorter length of the required pipeline sections up- and downstream of the orifice. The recommended upstream section length, after 90° turn, for a single-hole orifice [10] can exceed 40 duct diameters, while using a multi-hole orifice could allow for accurate mass flow measurement with a shorter duct length. Due to this, multi-hole orifice flow meters can be used on existing installations where the space is limited.

According to the Bernoulli and continuity equations, the pressure drop on the orifice can be used for mass flow estimation when the duct geometry and fluid density is known. Combining these two equations leads to the following relation for mass flow rate:

(1) $$q_{m}=\frac{1}{\sqrt{1-\beta^{4}}}\frac{\pi }{4}d^{2}\sqrt{2\Delta p\rho_{1}}$$

Contraction coefficient *β* is the square root of orifice to duct cross-section area ratio,

(2) $$\beta =\sqrt{\frac{A_{or}}{A_{d}}}=\frac{d}{D}$$

The contraction coefficient is the most important parameter describing the orifice. According to Equation (1), there is a strong dependency between the mass flow rate and contraction coefficient. What is more, this parameter is also used to determine flow coefficient and expansion number in further calculations, Equations (4) and (5). 

However, Equation (1) is valid only for the laminar flow of an incompressible, frictionless fluid in a horizontal channel. In practice, there are no ideal fluids and some corrections have to be introduced to the Equation (1) in order to perform correct flow measurement. Such an approach is presented in the ISO standard [10] where the equation for fluid mass flow determination is in the following form, with the flow coefficient and expansion number added:

(3) $$q_{m}=\frac{C}{\sqrt{1-\beta^{4}}}\varepsilon \frac{\pi }{4}d^{2}\sqrt{2\Delta p\rho_{1}}$$

The flow coefficient C and expansion number ε are calculated from [1,2]:

(4) $$C=0.5961+0.0261\beta^{2}-0.216\beta^{8}+0.000521{\left(\frac{10^{6}\beta }{Re}\right)}^{0.7}+\;\left(0.0188+0.0063{\left(\frac{19000\beta }{Re}\right)}^{0.8}\right)\beta^{3.5}{\left(\frac{10^{6}}{Re}\right)}^{0.3}$$

(5) $$\varepsilon =1-\left(0.351+0.256\beta^{4}+0.93\beta^{8}\right)\left[1-{\left(\frac{p_{2}}{p_{1}}\right)}^{\frac{1}{\kappa }}\right]$$

The calculation procedure described in ISO 5167 can be used in the limited range of Reynolds numbers (based on the channel diameter) and pressure ratios,

$$Re_{D}>5000\;if\;0.1\leq \beta \leq 0.56\;Re_{D}\geq 16000\beta^{2}\;if\;\beta >0.56\;\frac{p_{2}}{p_{1}}\geq 0.75$$

However, the Formulas (3)–(5) have not been developed and generalized for the multi-hole orifice with dimensions presented in this paper.

The aim of this paper is to investigate the flow through a six-hole orifice flow meter and to study the influence of orifice geometry on flow coefficient, then analyze the impact of flow disturbance on measured pressure drop. To assess the functionality of six-hole orifice as a flow meter, four geometrical variants were tested experimentally and numerically. The results were compared with standard single-hole orifices of the same contraction coefficient. The differences in flow coefficients and pressure drop coefficients were determined for both types of orifice in a wide range of Reynolds numbers. The effect of an obstacle partially plugging the flow upstream of the six-hole orifice was examined experimentally and numerically in terms of induced pressure drop difference.

### 1.1. Overview of Published Experimental Research

Experimental investigation of multi-hole orifice flow meters is usually focused on flow or pressure loss coefficient determination for different orifice configurations and contraction coefficients. 

Malavasi et al. [11] presented experimental results of pressure loss coefficient values for a wide range of multi-hole orifice flow meters. In total, 21 different orifice flow meters were investigated with a contraction coefficient ranging from 0.2 to 0.72 and for a number of orifice holes from 3 up to 52. Measurements were performed with water as a working fluid on two separate test stands. In the first one, the orifices were mounted inside test duct with 3” diameter. Pressure was probed at locations 2D upstream and 8D downstream the orifice. The other measurements were performed on 8” duct and pressure was probed 1D upstream and 10D downstream the orifice. Malavasi et al. [11] compared several multi-hole orifices with the same contraction coefficient and different number of holes. In their paper, it was noted that the average value of pressure loss coefficient *ξ* depends on the contraction coefficient. The results achieved by Malavasi et al. [11] are compared with other correlations and experimental data from scientific literature. Contraction of the orifice has dominant impact on *ξ* value but there were also other dependencies observed. In their experimental data the impact of orifice thickness can also be seen.

Pressure loss coefficient was also measured by Özahi [12]. In his research, pressurized dry air was used as the working fluid. The air was forced to the duct with D = 26.6 mm internal diameter and the length of 263 D (7 m). Investigated orifices were situated 188 D downstream the duct inlet and the pressure was probed 1D upstream and downstream of the orifice. The measurement of flow velocity was performed using the thermo-anemometric method at 50D distance from the inlet. Five kinds of orifice with a thickness of 3 mm and number of holes of 5, 9, 13 and 26, were used. Two examples of a 13-hole flow meter with different hole distribution were investigated. For all of the orifices investigated, the ratio of thickness to hydraulic diameter was constant and the contraction coefficient varied from 0.252 to 0.575. Pressure losses were measured in a turbulent flow regime in the duct with Reynolds numbers from 2500 to 9500. Pressure losses detected by Özahi [12] are consistent with other researchers. For the two 13-hole orifices, no significant differences in pressure loss coefficient were detected. On the basis of his experimental results, Özahi [12] proposed a simple correlation between contraction coefficient and pressure loss coefficient, which accuracy (standard deviation) is within 12%.

Huang et al. (2013) [13] published experimental results of the flow coefficient C obtained for multi-hole orifice flow meters, which were compared with the values measured for single-hole orifices. The measurements of flow coefficient were performed for orifices with hole number from 4 to 25, sharp edges, and 12 types of different arrangement. The contraction coefficient varied from 0.338–0.668. Most of the orifices had the same external diameter and thickness. Each multi-hole orifice was compared with a single-hole orifice of the same thickness and contraction coefficient. The authors performed flow measurements through the aforementioned orifices after mounting the orifices in a duct with diameter of D = 29 mm. The length of straight duct sections before and after the orifice was 2 m. The working fluid was water in normal conditions. The pressure difference was measured with the use of two methods: (1) immediately upstream and downstream the orifice and (2) 6D upstream and 1D downstream the orifice. The analysis of Huang et al. [13] focused on the dependence of the flow coefficient C on the orifice thickness, contraction coefficient, hole diameters and their distribution. What is more, the resistance to flow disturbance was also investigated. For this purpose, the 3D cross was mounted in the duct at the distance 15D, 10D and 5D upstream of the orifice. The results show that multi-hole orifices investigated by Huang et al. [13] have higher values of the flow coefficient than equivalent single-hole orifices. They are also characterized by lower critical Reynolds number, above which the flow coefficient value is constant. The authors noted the impact of multi-hole orifice thickness on flow coefficient. Increasing the thickness initially caused the increase of the flow coefficient but, above a certain value of the thickness, the flow coefficient remained constant for supercritical values of Reynolds number. Huang et al. [13] explained this as a result of friction forces increase in long channels. Their studies of the contraction coefficient effect on the flow coefficient did not return consistent results. Although the positive impact on flow coefficient was observed for the orifices of 5 mm diameter, the tendency for orifices of 3 mm diameter was reversed.

The influence of a hole diameter under constant contraction coefficient and thickness was determined by Huang et al. [13] on the basis of a comparison of three orifices. It was shown that the hole diameter has impact on the critical value of Reynolds number and the value of C in the supercritical Reynolds number region. The best results were achieved for the middle diameter value of 4 mm, where the discharge coefficient reached the highest value. It should be marked that the three orifices used in this analysis had a different number of holes and also arrangement. However, the authors indicated that the hole configuration should not have significant impact on critical Reynolds number and subcritical C value. Such conclusion results from comparison by Huang et al. [13] of two 6-hole orifices with the same contraction coefficient and different hole arrangement. The measured distributions of C(Re) function were similar. The resistance to flow disturbance of multi-hole orifices was investigated by Huang et al. [13] on the basis of a flow coefficient measurement for three orifices and compared with the results achieved for equivalent single-hole orifices. The flow disturbance was generated by the cross placed at the distance 15D, 10D and 5D upstream of the orifice. The following orifices were investigated: No. 1 (6 holes on a circle 0.69D, contraction β = 0.422), No. 2 (3 holes on a circle 0.37D + 8 holes on a circle 0.78D, β = 0.597) and No. 3 (5 holes on a circle 0.41D + 10 holes on a circle 0.86D, β = 0.668). All orifices had the same thickness of 3 mm with each hole diameter equal to 5 mm. The best resistance to disturbance had the No. 1 orifice, for which the flow coefficient decreased by only 2.6% for the case with the obstacle placed 5 D upstream the orifice. For the rest of the multi-hole orifices, the flow coefficient decreased more and more with the obstacle being bring closer to the orifice and these multi-hole orifices had similar resistance to disturbance to the conventional single-hole orifices. 

Zhao et al. [14] classified multi-hole orifices in terms of their geometrical parameters and then investigated the change of pressure loss coefficient for variable Reynolds number for orifices of different classes. The authors compared these results with measurements of equivalent single-hole orifice and proposed a correlation for pressure loss coefficient *ξ*. All multi-hole orifices investigated by Zhao et al. [14] had the thickness of 2 mm and each hole had the same diameter but their number varied from 3 to 13. The orifice parameters were measured on a horizontal duct of 6 m length and internal diameter D = 50 mm. The working fluid was demineralized water and the range of investigated velocity was from 0.1 to 1 m/s. Upstream pressure was calculated as the average from pressure values probed at distances 12 D, 9 D and 6 D from the orifice. The pressure on the downstream side was calculated in a similar way with the values probed 20 D, 24 D and 28 D from the orifice. Furthermore, the pressure was also probed at locations 1 D upstream and 0.5 D downstream the orifice, which allowed comparative calculations compatible with ISO 5167 standard. In the first part of their study, Zhao et al. [14] analyzed the effect of distribution and a number of holes on the value of pressure loss coefficient. It was found that the *ξ* values of the orifices’ nozzles differ the most for small Reynolds numbers. These differences decrease as the Reynolds number increases. Above the Reynolds number of 15,000, the losses for 3-, 5- and 6-hole orifices were similar to those in the standard (1-hole) orifice, while the losses in the 9- and 13-hole orifices were lower. The second part of the study focused on orifices with three selected patterns of hole placement. The orifice of each pattern was made in three versions, differing in the diameter of the holes. Zhao et al. [14] found that contraction is the dominant parameter affecting pressure loss in a multi-hole orifice. They developed a correlation that defines pressure loss coefficient as a function of contraction coefficient. What is more, they also noticed that the Reader–Harris/ Gallagher formula for calculating the flow coefficient in standard orifices according to ISO 5167 can be successfully applied to the multi-hole orifices they tested.

Đurđević et al. [5] investigated pressure loss coefficient for several types of multi-hole orifice in terms of Reynolds number. The working fluid was humid air with pressure from 2 bar to 16 bar. The results for 9-hole orifices were compared to the results for a single-hole orifice with equivalent contraction coefficient. It turned out that significant decrease in singular pressure loss coefficient was recorded for MHO compared to SHO and that MHO hole distribution had no significant influence to singular pressure loss coefficient. What is more, according to the authors compared to SHO, MHO was less responsive to flow change.

In addition to the publications discussed above considering experimental investigations of orifices with circular holes, similar works can be found regarding multi-gap orifices, i.e., with rectangular holes. For example, Morrison et al. [15] published an analysis of measurement of the flow of a water–air mixture through such an orifice. Geng et al. [16] investigated a moist gas meter using, among others, two orifices and a neural network. Li et al. [17] dealt with the problems of calibration of the multi-gap orifice for measuring the amount of wet gas. The research words discussed above are summarized in Table 1 with the specified scope of the work as well as the experiment details. The present study has also been added to the table.

### 1.2. Overview of Published Numerical Research

There are many results of numerical flow simulations through multi-hole orifice flow meters reported in scientific literature [8,16,18,19]. Most publications are focused on the accurate modelling of fluid flow in adopted geometry and optimization of the orifice arrangement and its profile. 

The results of CFD (computationa fluid dynamics) calculations for seven cases of 9-hole orifice flow meter were published by Singh and Tharakan [3]. All variants of the investigated geometries had duct diameter D = 21.2 mm and contraction coefficient equal to β = 0.5, eight peripheral holes arranged on a circle and one central hole. The differences between individual orifices were in the hole arrangement and the relation of central hole diameter to the diameter of the other holes. For comparison, the flow simulation through a single-hole orifice had also been performed. A standard orifice and two versions of multi-hole orifices were also tested experimentally. The experiment showed approximately 7% difference in pressure drop compared with CFD. The pressure drop obtained from numerical analysis allowed the discharge coefficient to be calculated,

(6) $$C_{D}=\frac{q_{m}}{\frac{\pi }{4}d^{2}\sqrt{2\Delta p\rho_{1}}}=\frac{C}{\sqrt{1-\beta^{4}}}\varepsilon$$

The numerical results showed that the discharge coefficient for SHO has the lowest value among all of the tested orifices. There were also differences in discharge coefficient identified for the investigated MHOs with the same contraction coefficient and different hole arrangement. 

Similar analysis was also performed by Yu et al. [20] The researchers focused mainly on checking the consistency of various numerical approaches with experiments.

Shaaban [4] made a series of CFD calculations of flow through 4-hole orifice in search of minimum loss of the pressure. Each hole had the shape of a convergent–divergent nozzle. The aim of his research was to find the optimal angles on both parts of the nozzle. Pressure drop in an orifice with cylindrical holes was also calculated in order to compare the results. The author performed the analysis for the convergent angles in the range from 0° to 60°, and from 0° to 15° for the divergent part. According to his results, the optimal angles are 50° and 7° for the convergent and divergent parts, respectively, and their impact on the pressure drop is significant. 

Geng et al. [16] and Kumar and Bing [8] undertook similar numerical simulations as described above, but they investigated mainly multi-gap orifices with rectangular holes. Geng et al. [16] chose the orifice with contraction coefficient of β = 0.5 to their flow simulations. In this orifice, there were 48 rectangular holes measuring 2 × 5.32 mm, placed on concentric circles. Part of the calculations was done for the dry air flow and compared with the results for equivalent conventional orifice. The results showed that the distortion of pressure and velocity field vanished quickly in the orifice with 48 gaps, while in the case of the conventional orifice this process was much slower. Kumar and Bing [8] considered several versions of multi-gap orifice or multi-hole orifice to measure humid air flow. In this case, the authors also used single-hole orifice as the reference. All orifices had the same contraction coefficient β = 0.40 and were designed for the duct of 105.74 mm internal diameter. 

In the present paper the flow coefficient C from Equations (3), (4) and (6) is analyzed. The paper presents the experimental results from measurements on a test stand as well as the results of numerical simulations of an ideal gas flow through a multi-hole orifice flow meter, which is mounted in a circular pipe. The analysis was performed for several orifice geometries defined in Table 2. In the first part of the analysis, experimental measurement of the flow coefficient has been performed on several six-hole orifices with the contraction coefficient value from 0.5164 to 0.7. The methodology for these measurements was developed and high accuracy differential pressure meters were used. The impact of chamfered orifice edges on the flow coefficient was also investigated. Then, in the second part, the effect of a non-uniform flow field on the pressure drop has been analyzed experimentally. An obstacle has been introduced to the duct, upstream of the orifice, which generated a disturbed inflow on the orifice. The results show the pressure difference and the potential error in mass flow estimation for such cases. In the third, final part, the additional numerical simulations have been performed for the case of six-hole orifice and disturbed flow. The purpose of the numerical part is to show the fluid flow patterns near the orifice and develop a numerical tool for pressure drop prediction in similar cases. Investigation of the non-uniform velocity field effect on the pressure drop measured on the orifice can be treated as the original novelty of this article, because it is not a popular subject in scientific literature and such papers can hardly be found. What is more, the paper concerns orifice geometries, which are not featured in available literature, and presents a comprehensive approach to the topic. 

## 2. Materials and Methods

**Figure 1:**
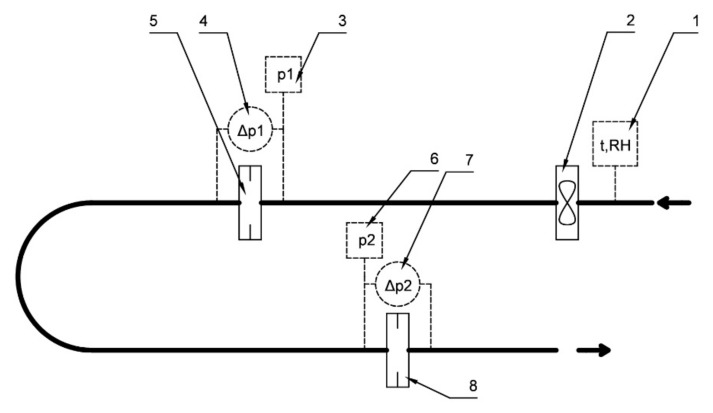
The experimental setup.

As a part of the present research, in order to investigate the relationship between pressure drop and mass flow, an experimental stand was designed and built (Figure 1). It included temperature and relative humidity meter (1), inlet fan (2), pressure sensors (3,6), differential pressure meters (4,7) and two orifice housings (5,8). The accuracy class of the differential pressure meters used (4,7) was 0.05% and 0.065%, respectively. Airflow was generated by an inlet fan, the rotation speed of which was controlled with an inverter. In this way the required mass flow rate was set. In the test stand, there were two orifice housings, in which different orifices could be mounted. The idea of the first part of the experiment was to measure the mass flow rate through the first, standardized single-hole orifice and then calculate, from formula (1), the flow coefficient value for the second orifice. Such a procedure is possible when the fluid density and viscosity upstream each orifice is known as well as the pressure drop on each orifice. In order to calculate these parameters, the temperature, relative humidity and total pressure were measured. The humid air density was calculated from simplified empirical correlations and treating water vapor and air as an ideal gas. The accuracy of the aforementioned correlations was compared with the model implemented in CoolProp software and for the experimental conditions it was at a desirable level.

(7) $$\rho_{air}=\frac{p-p_{v}}{R_{a}\cdot T_{a}}+\frac{p_{v}}{R_{v}\cdot T_{a}}$$

where water vapor partial pressure equals:

(8) $$p_{v}=\varphi \cdot 610.7\cdot 10^{\frac{t_{a}}{31.6885+0.130986t_{a}+2.52493\cdot 10^{-5}t_{a}^{2}}}$$

Humid air dynamic viscosity was calculated from linear interpolation of tabularized data. The procedure has been implemented in LabVIEW environment to process all measured signals. The data were acquired with the frequency of 1 kHz, averaged over one second interval and then saved to a text file.

### Investigated Orifices

**Figure 2:**
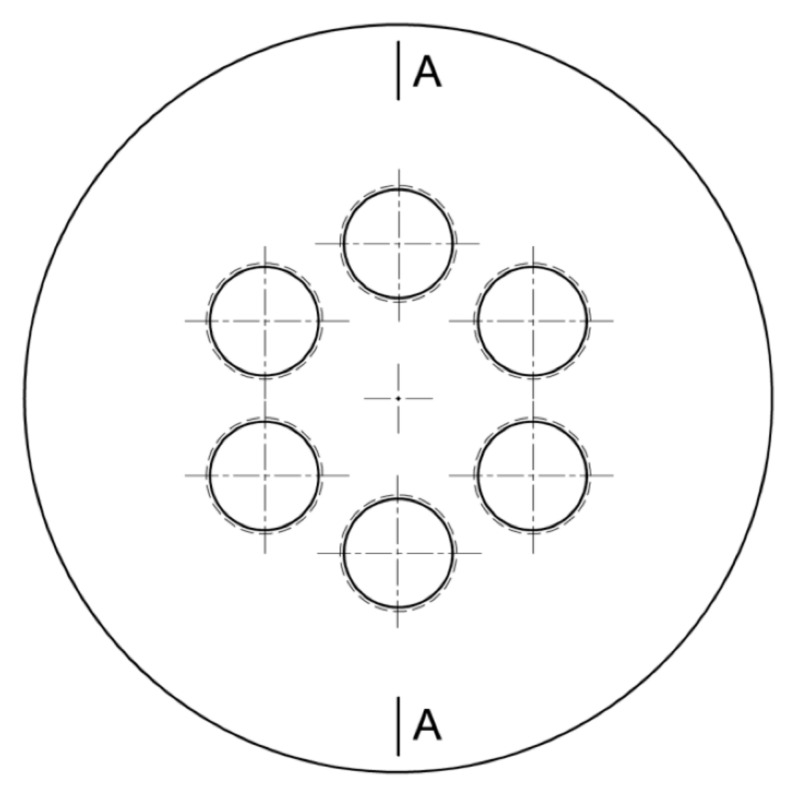
Front view of the multi-hole orifice.

**Figure 3:**
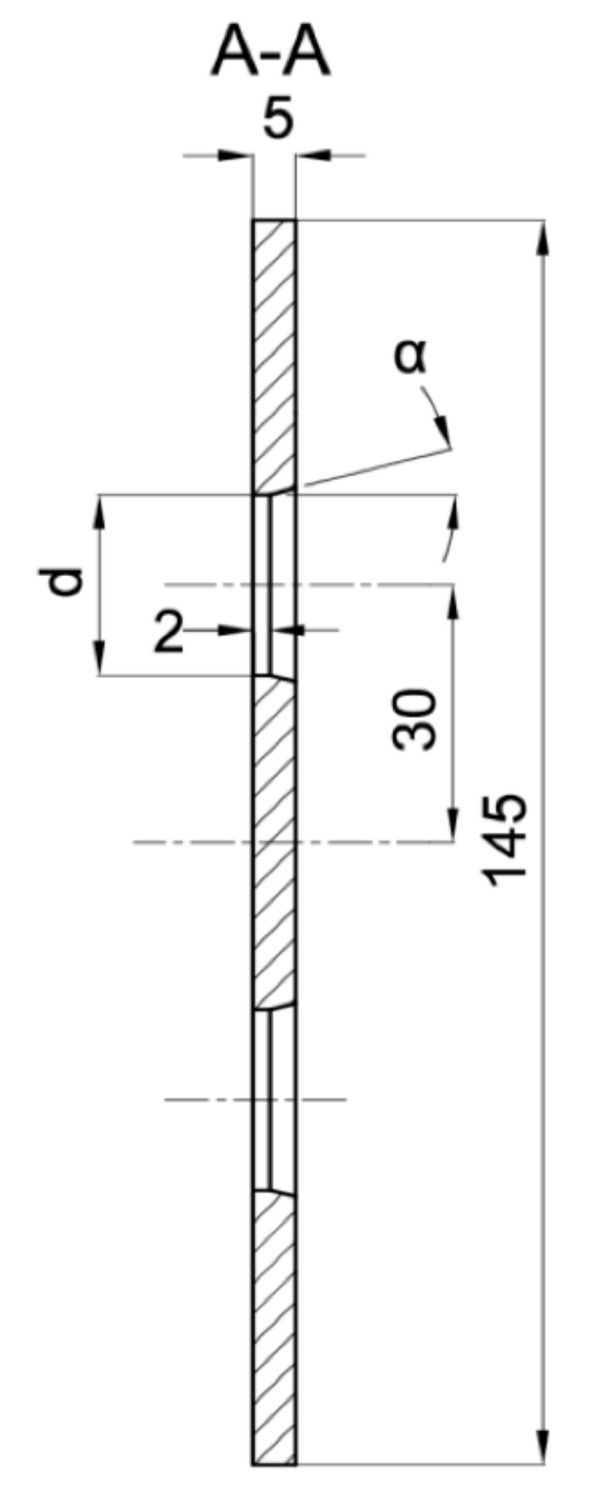
Hole profile.

During the research, several single- and multi-hole orifices were tested experimentally. All of them are listed in Table 2 with their exact dimensions. Six-hole orifices MHO1, MHO2 and MHO3 geometry is shown in Figure 2 and Figure 3. The orifice MHO4 with the biggest contraction coefficient had a different hole profile. It also the thickness of 5 mm but the edges were not chamfered due to the small distance between neighboring holes. Such a geometry difference can affect the pressure drop characteristics.

## 3. Results

The analysis presented in this paper can be divided into several separate parts. In the first section there is an experimental analysis of multi-hole orifice flow coefficient. Then, in the second part the impact of the flow disturbance on pressure drop is investigated experimentally and numerically. Their interpretation as well as the experimental conclusions that can be drawn follow.

### 3.1. Results of Flow Coefficient Measurements

**Figure 4:**
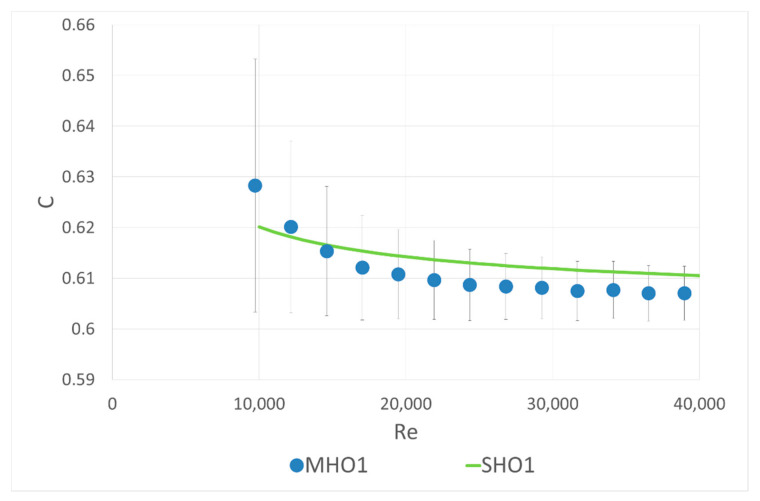
Comparison of flow coefficients for the single-hole orifice SHO1 and multi-hole orifice MHO1 of the same contraction coefficient β = 0.5164.

**Figure 5:**
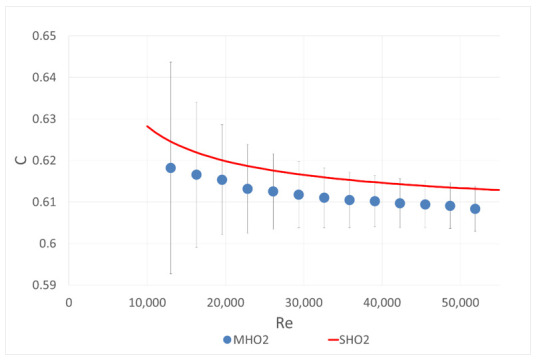
Comparison of the flow coefficients for SHO2 and MHO2 with the same contraction coefficient β = 0.6.

**Figure 6:**
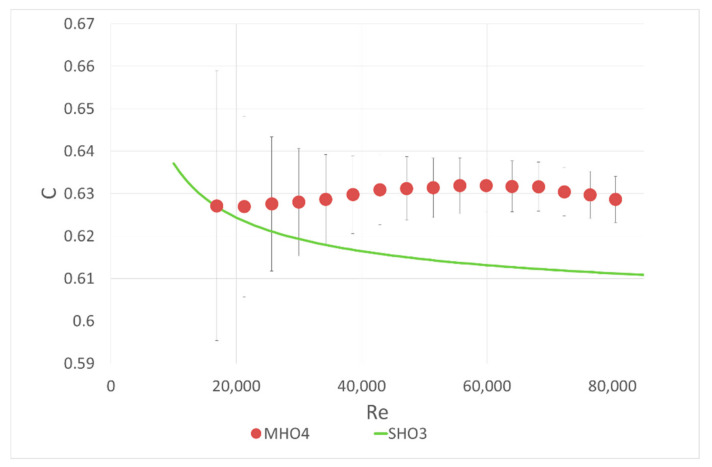
Comparison of the flow coefficients for SHO3 and MHO4 with the same contraction coefficient β = 0.7.

The idea of the first part of the experiment was as follows: a single-hole orifice was mounted in the first orifice housing and a 6-hole orifice was located in the second. The mass flow rate was measured by the single-hole orifice. With the known flow rate, the flow coefficient C included in Equation (1) was calculated for the investigated six-hole orifice. The calculated coefficients were compared with theoretical curve calculated with the correlation (2) from ISO 5167 [10] for a single-hole orifice with the same contraction coefficient. The results are presented in Figure 4, Figure 5 and Figure 6. Both mounted orifices had the same contraction coefficient so the pressure drop could also be directly compared.

The values obtained for the flow coefficient of MHO1 and MHO2 are slightly lower compared to SHO1 and SHO2, respectively. However, all measured differences between SHO and MHO are within the limits of the measurement error.

In the case of MHO4, the results show a different tendency than for the orifices with lower contraction coefficients. In this case the flow coefficient values for 6-hole orifice are higher than for the single-hole orifice, in particular for large Reynolds numbers, Figure 6. Such a tendency can be an effect of the MHO4 geometry, which was different to other investigated multi-hole orifices. The holes in each analyzed MHO were distributed on a circle of 60 mm diameter and in the MHO4 orifice, with the biggest contraction coefficient, the distances between neighboring holes were too small to make the chamfer process possible. For this reason, MHO4 had holes with sharp edges on both plate sides but according to further results it should not make any noticeable difference. Similar to the present results, Huang et al. [13] also observed different tendencies in flow coefficient for different hole distribution.

**Figure 7:**
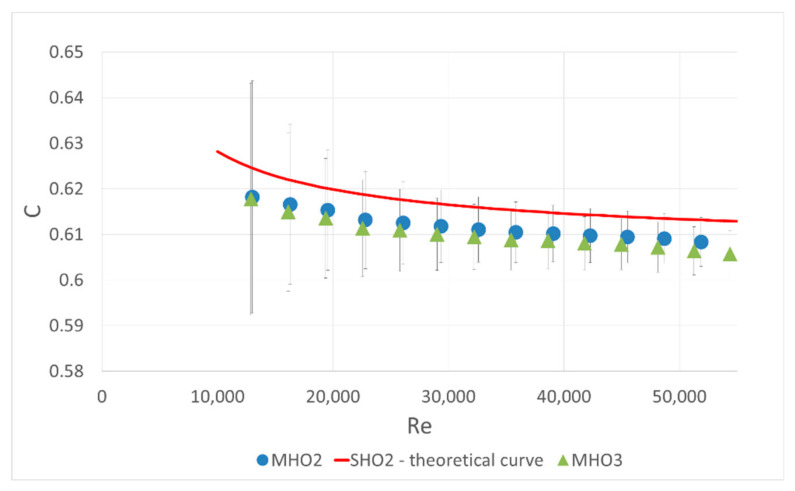
Experimental results for two multi-hole orifices with different chamfer angle and the same contraction coefficient β = 0.6.

During the experimental research several orifice geometries were tested. This included two orifices MHO2 and MHO3 with the same contraction coefficient but with a different angle of chamfer, 15° and 45° respectively. The results of their C coefficient comparison are depicted in Figure 7. Differences are not significant, which means that the chamfer angle has a negligible effect on the pressure drop. Such measurements also proved the repeatability of the experiment. 

In order to compare all orifices, pressure drop coefficient *ξ* was calculated. It is defined as a ratio of the actual pressure loss to the dynamic pressure in the orifice hole,

(9) $$\xi =\frac{\Delta p}{0.5\rho_{or}w_{or}^{2}}$$

**Figure 8:**
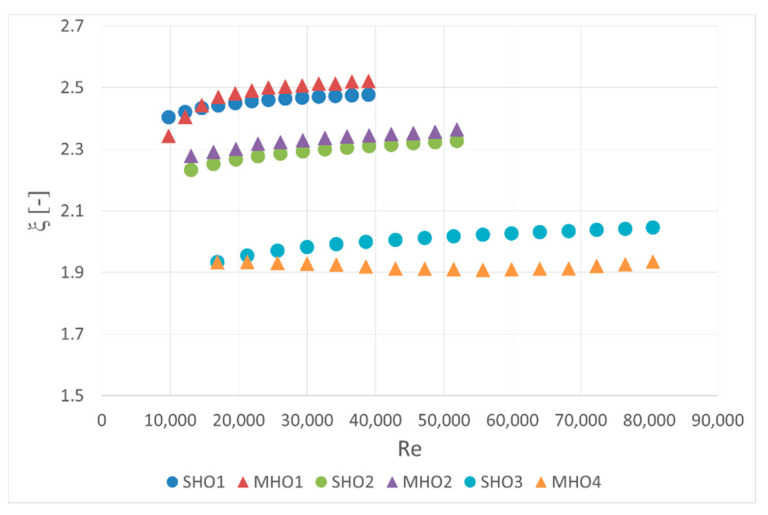
Pressure loss coefficient.

The fluid density was assumed to be constant and, therefore, it was calculated for the pressure upstream the orifice. The flow velocity was then calculated from the continuity equation. The results are summarized in Figure 8. The differences between single-hole and multi-hole orifices with the same contraction coefficient are marginal. This can be due to the absence of a central hole in the multi-hole orifices that, according to results reported by Đurđević et al. [5], can significantly decrease the value of pressure loss coefficient. 

**Figure 9:**
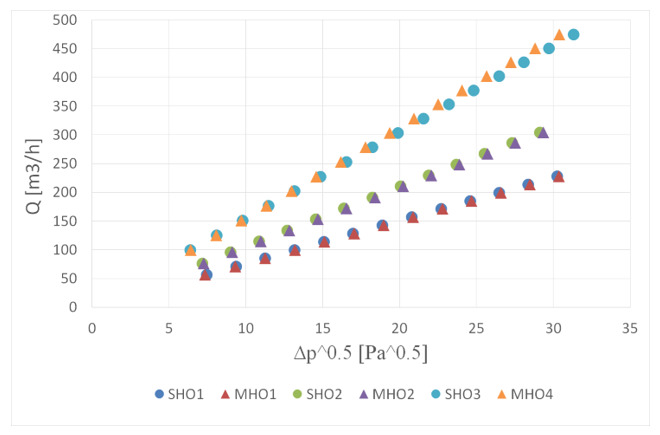
Volumetric flow rate versus pressure drop.

The relationship between volumetric flow rate and a square root from the measured pressure drop was also investigated. The results are presented in Figure 9. All orifices showed a linear relationship, however the differences between multi-hole orifices and the single-hole orifice can hardly be observed if the contraction coefficient is the same.

### 3.2. The Effect of Flow Disturbance on Orifice Pressure Drop

**Figure 10:**
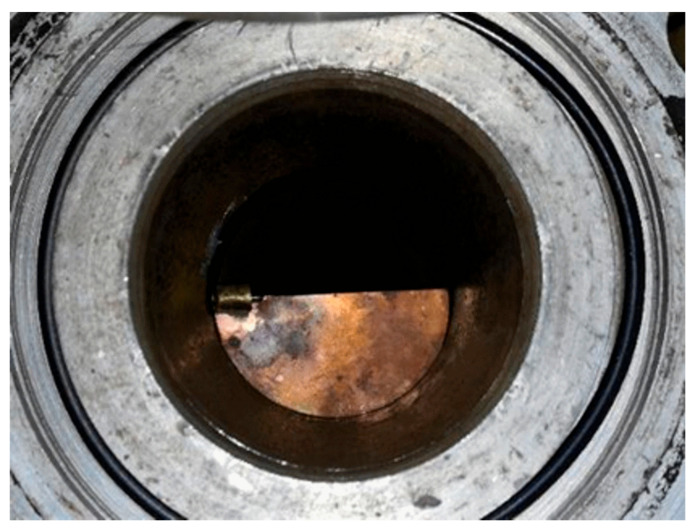
The obstacle placed in the duct.

The ISO 5167 standard requires straight duct lengths upstream and downstream of the single-hole orifice from 90° turn or similar obstacle. These distances increase with the value of contraction coefficient and can reach more than 40 D for the single-hole orifice with β = 0.7. The aim of the second part of the experimental research was to investigate the effect of non-uniform velocity profile in the upstream section of the orifice on the measured pressure drop value. The experimental stand remained almost unchanged. The only modification was the addition of an obstacle upstream the first orifice casing (Figure 10) in order to achieve significant flow disturbance upstream the orifice.

**Figure 11:**
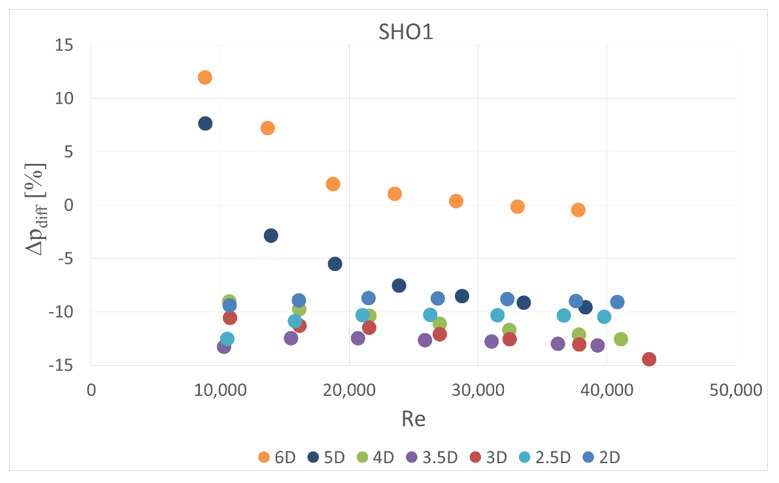
Pressure drop difference for SHO1.

**Figure 12:**
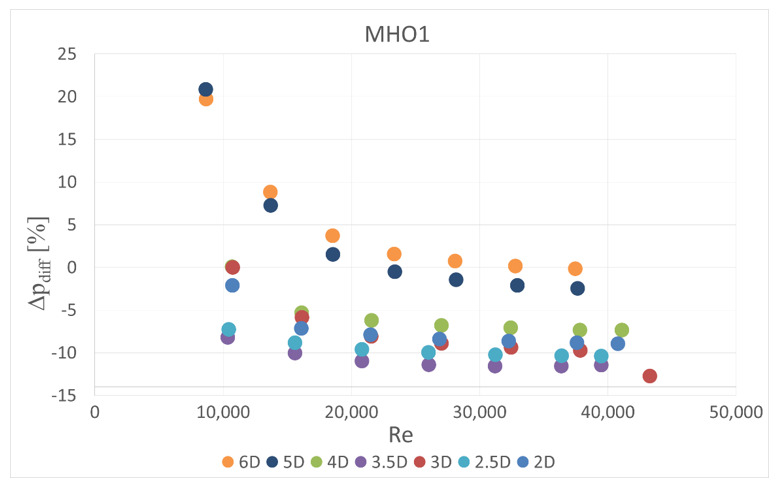
Pressure drop difference for MHO1.

**Figure 13:**
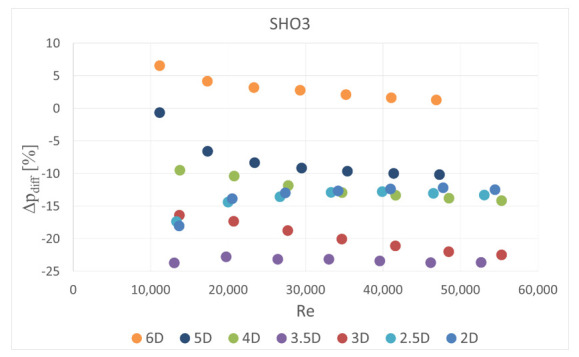
Pressure drop difference for SHO3.

**Figure 14:**
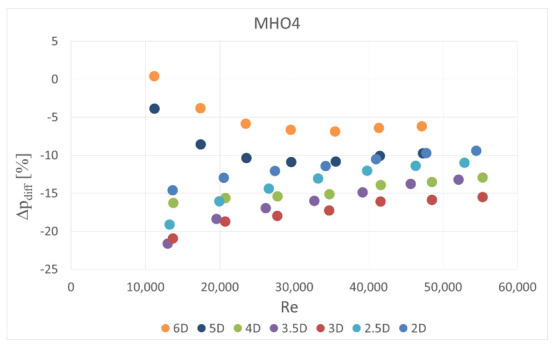
Pressure drop difference for MHO4.

The obstacle covered half of the channel cross-section. It was situated in five different locations—2 D, 2.5 D, 3 D, 3.5 D and 4 D, 5 D and 6 D upstream the orifice. In the first orifice casing there were mounted single- or six-hole orifices while in the second casing the reference mass flow measurement was carried out with the use of SHO2. Orifices with the lowest and highest contraction coefficient (SHO1, MHO1, SHO3, MHO4) were examined. The measured pressure drop was compared with the calculated reference pressure drop value based on the previous measurements with no obstacle and actual mass flow rate from the reference SHO2. The results of relative pressure drop differences $\Delta p_{diff}$ for several obstacle locations are presented in Figure 11, Figure 12, Figure 13 and Figure 14.

(10) $$\Delta p_{diff}=100\%\frac{\Delta p_{meas}-\Delta p_{ref}}{\Delta p_{ref}}$$

The reference pressure drop $\Delta p_{ref}$ was calculated from Equation (3) for the same Reynolds number (compared to the case with disturbed flow) and other flow parameters. The actual mass flow rate was measured on the second orifice. In the case of multi-hole orifices the flow coefficient was taken from experimental results (Figure 4, Figure 5 and Figure 6), while for single-hole orifices the flow coefficient was calculated from Equation (4).

**Figure 15:**
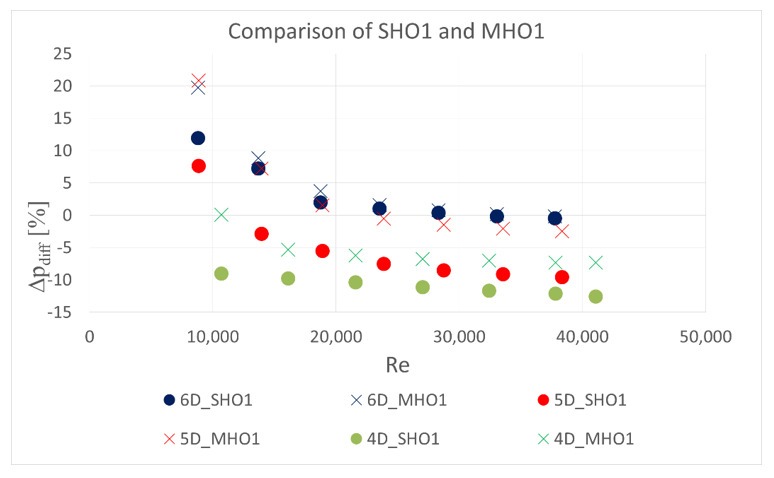
Comparison of multi-hole orifice and single-hole orifice with contraction coefficient of 0.5164.

The results showed that the examined multi-hole orifices with lower contraction coefficient had slightly better resistance to the flow disturbance than conventional single-hole orifices (see Figure 15). For some cases the pressure drop difference for a single-hole orifice was few times greater than for a six-hole orifice. Unexpectedly, it was observed that an obstacle at the distance of 3.5 D upstream from the orifice usually causes similar difference of measured pressure drop like for the distances of 2 D, 2.5 D and 3 D. Differences detected in pressure drop between the cases with and without flow disturbance show that the pressure drop is lower in the flow with the obstacle. Such a tendency would result in a higher discharge coefficient for both MHOs and SHOs. For the obstacle placed 5 D and 4 D upstream the orifice, MHO1 has noticeably better resistance to flow disturbance than SHO1. For the 6 D location the results are similar, while for the lower distances, flow seems to be strongly turbulent and the pressure drop is significantly altered on both orifices. For higher values of contraction coefficient, relative pressure differences are higher for both SHO3 and MHO4. It means that resistance to disturbance should decrease with an increase of contraction coefficient. For higher *β* ratios of 0.7, no significant differences were detected between SHO and MHO. It is possible that the flow non-uniformity was too high to detect differences in these kinds of orifice. 

**Figure 16:**
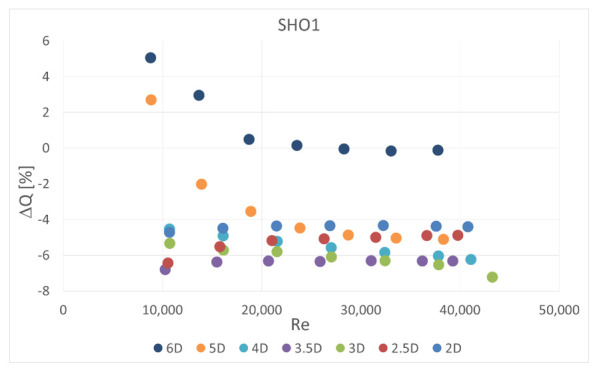
Measurement error of mass flow rate in SHO1 for disturbed flow.

**Figure 17:**
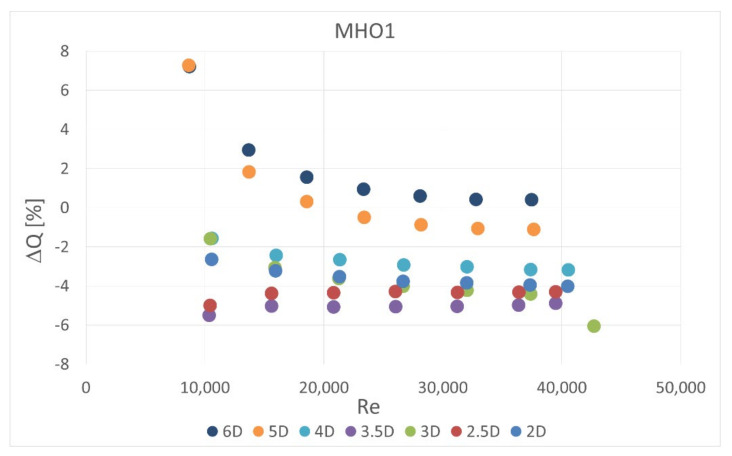
Measurement error of mass flow rate in MHO1 for disturbed flow.

**Figure 18:**
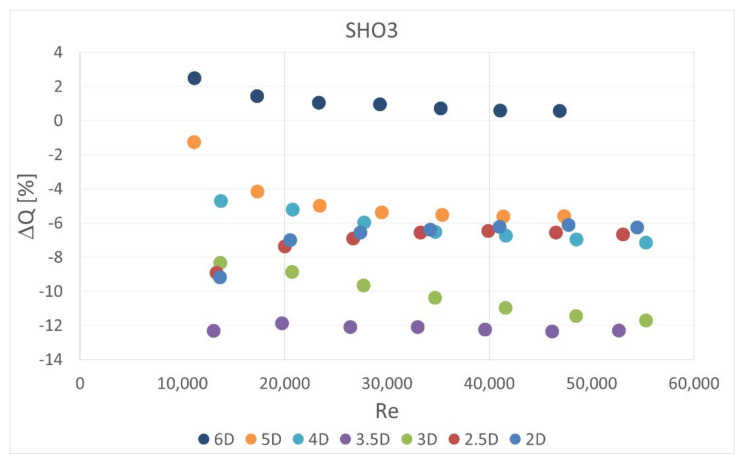
Measurement error of mass flow rate in SHO3 for disturbed flow.

**Figure 19:**
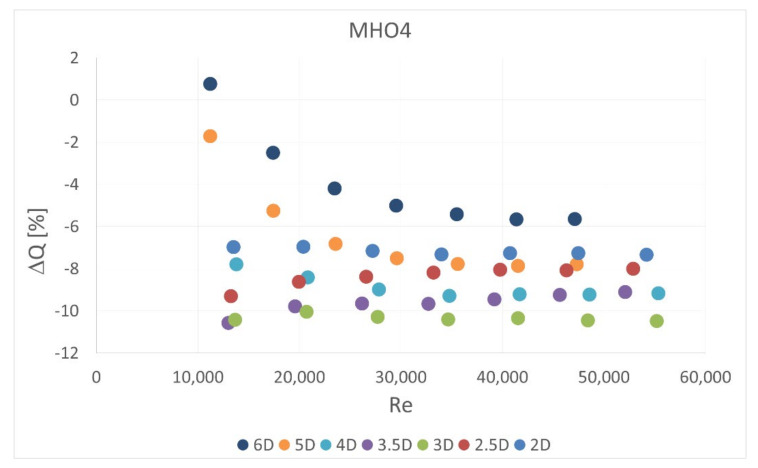
Measurement error of mass flow rate in MHO4 for disturbed flow.

The calculated mass flow rate, based on the measured pressure drop value for the case with an obstacle, was also compared with actual value measured simultaneously on the second orifice. The results are presented in Figure 16, Figure 17, Figure 18 and Figure 19. Mass flow measurement error was calculated from Equation (3) using the measured and reference pressure drops from Equation (10),

(11) $$\Delta Q=100\%\frac{\Delta Q_{meas}-\Delta Q_{ref}}{\Delta Q_{ref}}$$

Therefore the tendencies presented in the charts are similar. The flow measurement error is slightly lower in some cases for multi-hole orifices. Again, it can be observed that the obstacle placed 3.5 D upstream of the orifice led to the highest measurement error and smaller distances to the obstacle return similar results. In all of the analyzed cases the presence of the obstacle reduced the measured pressured drop on the orifice and, in consequence, the mass flow rate derived from it. The relative differences in measured mass flow rates were from 0% to 14%, depending on the case investigated.

### 3.3. Numerical Simulation Results

In order to further investigate the flow through the multi-hole orifice, a computational model was prepared and solved. The aim of this numerical analysis was to determine pressure drop differences caused by the presence of the obstacle and investigate the flow pattern in its proximity. 

**Figure 20:**
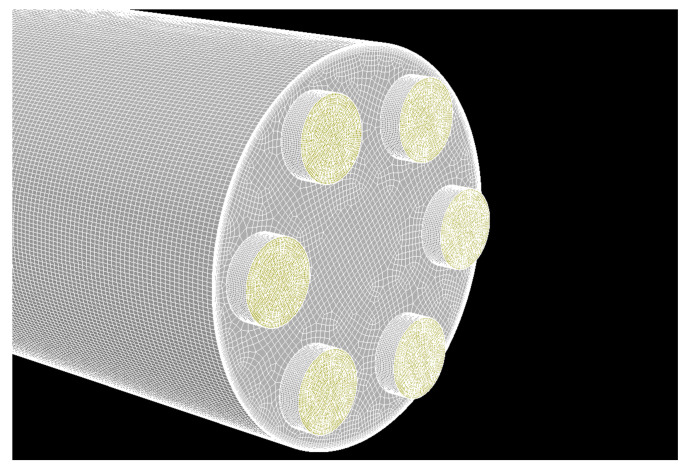
Close-up of the computational mesh (10 million elements in total) in the vicinity of orifice holes.

In the numerical simulation, the duct sections upstream and downstream of the orifice had a length of 10 D. Such dimensions should allow the flow to fully develop upstream of the orifice and should not affect the calculated pressure drop due to the fact that multi-hole orifice flow meters have better resistance to the flow disturbances compared to single-hole orifices. Therefore, in the simulation, longer duct length was not required. The computational domain does not have a symmetry plane due to the disturbance introduced to the flow. The domain has been discretized to over 10 million hexahedral elements. In order to properly model the flow in the boundary layer, 15 inflation layers were created on the duct wall and 7 on each hole perimeter. The thickness of the first layer was chosen to satisfy the condition of Y+ = 3 for expected flow velocity in the duct. Y+ is a dimensionless parameter describing the cell height in the y direction and it is a function of the Reynolds number. Although the value of Y+ = 1 is recommended while using the enhanced wall treatment model, it would result in a mesh with high aspect ratio elements and additional cells. The value used during mesh generation is a good compromise between computational effort, quick convergence and the accuracy of the results. The whole computational domain was divided into 10 parts in order to perform proper discretization process. All segments were then combined with mesh interfaces. The grid was refined in the proximity of each hole (Figure 20).

All of the computational cases were carried out with absolute outlet pressure equal to 101.13 kPa and inlet temperature of 15 °C. Numerical simulation was performed by solving the steady state Reynolds averaged Navier–Stokes equations (RANS). The realizable k–ε turbulence model with an enhanced wall treatment option enabled was used in the calculations, which according to Shaaban [4] and Mehmood [6] should lead to proper results. The following discretization schemes were used: second order scheme for pressure, SIMPLE scheme for pressure–velocity coupling and second order upwind scheme for momentum, kinetic energy and turbulence dissipation rate. The gas flowing through the orifice was dry air, the density of which was described by the ideal gas law. The boundary conditions used in the simulations were mass flow inlet and pressure outlet.

**Figure 21:**
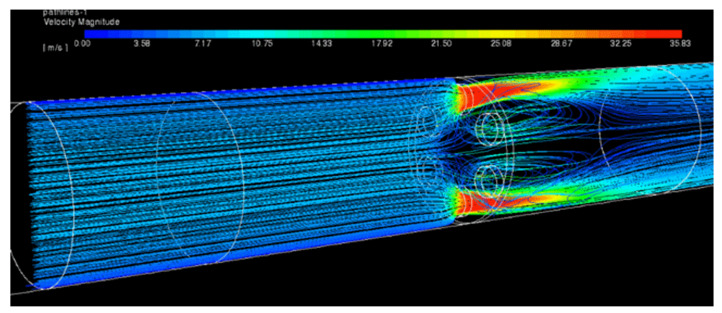
Streamlines for the reference case displayed on horizontal cross-section.

**Figure 22:**
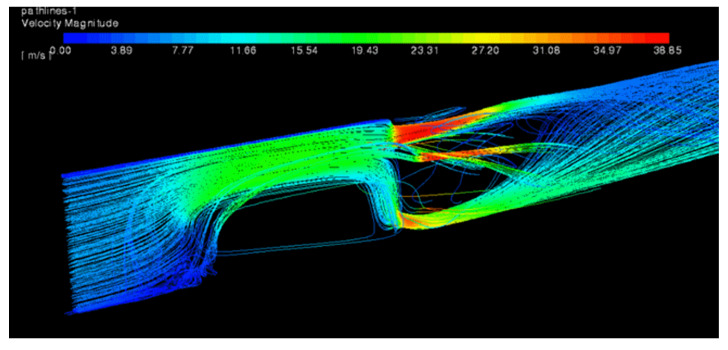
Streamlines for the case with disturbed flow displayed on horizontal cross-section.

**Figure 23:**
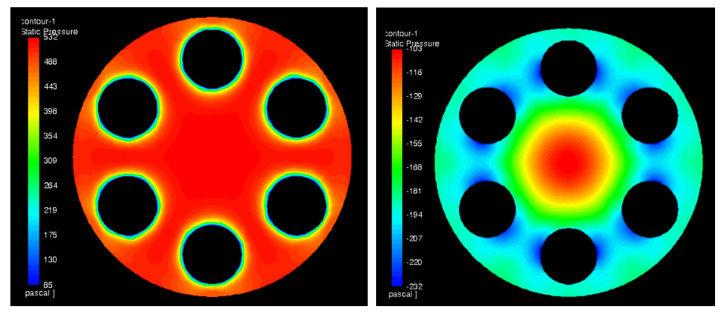
Pressure drop distribution for the case without the obstacle on the upstream (**left**) and downstream (**right**) side of the orifice.

**Figure 24:**
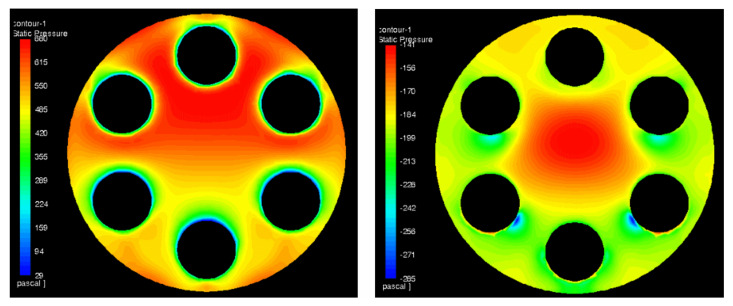
Pressure drop distribution for the case with the obstacle on the upstream (**left**) and downstream (**right**) side of orifice.

The calculations were performed for MHO1. There were two computational cases prepared. The first one—a reference case with no obstacle—and the second one with half of the duct blocked at location 3 D upstream the orifice. The aim of the numerical analysis was to study the influence of the disturbed flow on the pressure drop and to visualize the recirculation zone as well as other flow patterns near the obstacle. Figure 21 presents streamlines, colored by velocity, for the reference case and Figure 22 for the case with the obstacle. The streamlines were released from a narrow, vertical surface (at the left side of the images) in order to reduce its number and obtain a clear image of the flow patterns. Due to this, most of the streamlines shown pass through two holes found on a plane coinciding with the streamlines origin but the whole flow is uniformly distributed among all holes in Figure 21. The pressure distribution on both sides of the orifice has also been depicted and it is shown in Figure 23 and Figure 24 for the case without and with the obstacle present, respectively.

**Figure 25:**
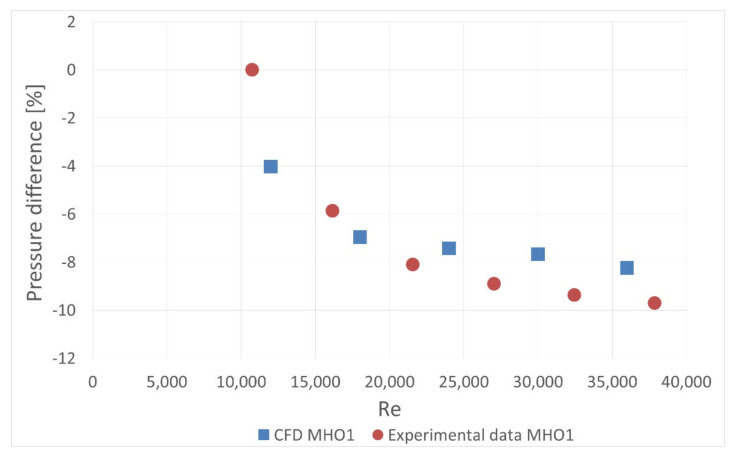
Pressure drop difference for the case with disturbed flow. Validation of the numerical model.

To determine the orifice pressure drop, the calculated pressure field was probed at six points along the duct perimeter immediately upstream and downstream of the orifice plate. The pressure difference was calculated and then the values obtained for disturbed and undisturbed flow were compared. The resulting pressure drop difference for MHO1 is presented in Figure 25. It is compared with experimental data in order to validate the model. The numerical results show good consistency with experiment, which proves that numerical approach can be successfully used to predict the effect of flow disturbance on pressure drop.

## 4. Discussion

The aim of this research was to investigate, experimentally and numerically, the relation between pressure drop and mass flow rate in multi-hole orifice flow meters. Several orifice geometries were tested in a wide range of Reynolds numbers. The experimental analysis showed that the empirical formula for pressure drop calculation in a single-hole orifice flow meter, included in the ISO 5167 standard [10], can also be successfully used for the investigated six-hole orifice flow meters. The relative difference between flow coefficient obtained from an experiment with multi-hole orifices and from ISO 5167 for single-hole orifices with the same contraction coefficient was within the measurement error range. According to Huang et al. [13] many factors, including the hole distribution, can influence the flow coefficient. The authors of [13] observed that a multi-hole orifice had sometimes higher and sometimes lower discharge coefficient than a conventional orifice with the same contraction coefficient. However, the differences detected by them were, for some orifice shapes, higher than presented in this article. Nevertheless, they did not examine the exact geometries as presented in this paper. Đurđević et al. [5,7] observed lower pressure loss coefficients for multi-hole orifice flowmeters but the orifices were distributed closer to the orifice center or there was an additional central hole, which probably made the pressure drop smaller. Özahi et al. [12] investigated the impact of contraction coefficient on pressure loss coefficient in perforated plates and proposed a simple correlation. The accuracy of this correlation is about 12% and it is a function of contraction coefficient only, so it follows that the differences between orifices with different hole number and their distribution should fall into this range of uncertainty.

In the present paper it was found that MHO1 and MHO2 have slightly lower flow coefficient than single-hole orifices but the differences detected are within the measurement error. By contrast, MHO4 has a higher flow coefficient than SHO3 but still the relative difference is lower than 5%. Such results are consistent with the results obtained by Huang et al. [13] and Özahi et al. [12]. However, measurements were performed in different conditions and it is hard to directly compare the results obtained. The research proved that flow coefficient for investigated geometries of multi-hole flow meters should be a function of contraction coefficient and Reynolds number. The effect of edges chamfer angle on the pressure drop was also examined and according to the results obtained it should be negligible for the examined orifice thickness. In addition, the effect of flow disturbance on pressure drop was investigated experimentally and numerically. Experimental analysis showed that examined six-hole orifice flow meters have in some cases better resistance to a non-uniform velocity field and their application in disturbed flow should lead to smaller measurement error compared to conventional orifices. Huang et al. [13] showed that perforated plates have higher discharge coefficients than single-hole orifices in a case for disturbed flow and indicated that multi-hole orifices have slightly better resistance to disturbance but there is no direct comparison presented in their paper. They also detected different trends in discharge coefficient for single-hole orifices and multi-hole orifices while altering the distance to the obstacle. 

In the present study, there were differences in pressure drop detected between the cases with and without flow disturbance. Introducing the obstacle lowered the measured pressure drop. For the obstacle located 5 D and 4 D upstream the orifice, MHO1 had significantly better resistance to flow disturbance than SHO1. For the 6 D distance, there was no differences between these two orifices, while for the shorter distances flow showed a strongly turbulent character and the pressure drop was significantly changed on both orifices compared to the reference values. For the contraction coefficient equal to 0.7, relative pressure differences were higher both for SHO3 and MHO4. There was a tendency for resistance to disturbance to reduce with an increase of contraction coefficient. For higher β ratios of 0.7, there were no significant differences detected between SHO and MHO. It is possible that the flow was too turbulent to detect differences in these kinds of orifice. Numerical simulations showed good agreement with experimental data for the relative pressure difference caused by the obstacle. The numerical model can be used for estimation of the flow disturbance effect on a pressure drop in similar cases.

## Figures and Tables

**Table 1:** Summary of experimental research regarding multi-hole orifices compared with the present study.

Author	β	Re	*D*	Number of Holes	Working Fluid	Investigated Relationship
Malavasi et al. [13]	0.2–0.72	5000 to 3 × 10^5^	3″ and 8″	3–52	water	*ξ* (Re), *ξ* (*t*)
Özahi [15]	0.252–0.573	2500 to 9500	26.6 mm	5–26	pressurized air	*ξ* (β), *ξ* (Re)
Huang et al. [14]	0.338–0.668	1000 to 4 × 10^4^	29 mm	4–25	water	C(*t*,Re), resistance to flow disturbance
Zhao et al. [16]	0.2–0.4	8000 to 2.5 × 10^4^	50 mm	3–13	demineralized water	*ξ* (Re)
Đurđević et al. [11]	0.5–0.7	1.5 × 10^4^ to 5.7 × 10^5^	70.3 mm	9	pressurized humid air	*ξ* (β), *ξ* (Re)
present study	0.5164–0.7	1 × 10^4^ to 8 × 10^5^	100 mm	6	humid air	C(Re), resistance to flow disturbance

**Table 2:** Examined geometries of flow meters

Flow Meter	Hole Diameter d [mm]	Contraction Coefficient β [-]	Chamfer Angle α [◦]
SHO1	51.64	0.5164	45
MHO1	21.082	0.5164	15
SHO2	60	0.6	45
MHO2	24.495	0.6	15
MHO3	24.495	0.6	45
SHO3	70	0.7	45
MHO4	28.58	0.7	0

## Nomenclature

$A_{d}$	duct cross-section area
$A_{or}$	orifice cross-section area
C	flow coefficient, empirical correlation proposed by Reader-Harris & Sattary [1]
$C_{D}$	discharge coefficient
D	duct diameter
d=βD	(equivalent) orifice diameter
$p_{1}$	absolute pressure upstream the orifice
$p_{2}$	absolute pressure downstream the orifice
$p_{v}$	water vapor partial pressure
Δp	pressure drop
$\Delta p_{ref}$	reference pressure drop for the case without obstacle
$\Delta p_{meas}$	measured pressure drop for the case with obstacle
$\Delta p_{diff}$	relative pressure drop difference as an effect of the obstacle
$q_{m}$	mass flow rate
$\Delta Q_{ref}$	reference mass flow rate calculated for the pressure drop for the case without obstacle
$\Delta Q_{meas}$	mass flow rate calculated for the measured pressure drop for the case with obstacle
ΔQ	relative mass flow difference as an effect of the obstacle
$R_{a}$	air gas constant
$R_{v}$	water vapor gas constant
$Re_{D}$	Reynolds number based on duct diameter
$t_{1}$	gas temperature upstream the orifice
$w_{or}$	gas velocity in the orifice hole
β	contraction coefficient
ε	expansion number defined by Reader-Harris [2]
κ	Poisson number
$\rho_{1}$	gas density upstream of the orifice
$\rho_{or}$	gas density in the orifice hole
ξ	pressure loss coefficient
φ	relative humidity
SHO	single-hole orifice
MHO	multi-hole orifice
